# A N-of-1 social network approach to study the social dynamics of alcohol consumption

**DOI:** 10.1080/21642850.2025.2465616

**Published:** 2025-03-09

**Authors:** Dominika Kwasnicka, Aileen O’Gorman, Martin Anderson, Louise Bowman, Mark McCann

**Affiliations:** a Melbourne School of Population and Global Health, University of Melbourne, Melbourne, Australia; b School of Education and Social Sciences, University of the West of Scotland, Paisley, Scotland; c MRC/CSO Social and Public Health Sciences Unit, University of Glasgow, Glasgow, Scotland; d Scottish Drugs Forum, Glasgow, Scotland

**Keywords:** Alcohol, ecological momentary assessment, N-of-1, social dynamics, social network analysis‌

## Abstract

**Introduction:**

The aim of this study was to investigate how the dynamics of the social environment impacted the alcohol consumption of individuals who self-identified as heavy drinkers.

**Methods:**

A mixed methods approach including N-of-1 study with daily Ecological Momentary Assessment (EMA) followed by a social network egonet interview. Qualitative data was analysed using deductive and inductive approaches. The main quantitative outcomes were a number of social contacts and the supportiveness of social networks.

**Results:**

Fifteen participants provided sufficient EMA data regarding social contact and six of these took part in the egonet interviews. EMA respondents reported 10.8 social contacts on average and rated approximately half of their networks as positive supports; approximately 10% of each respondents’ networks were perceived as ‘drinking a lot’. Interview data illustrated the influence of peer and family networks; stress; motivation levels; and coping strategies within the context of the social world. EMA and egonet methods proved feasible with this specific population demonstrating the utility of innovative approaches to study dynamic social contexts related to substance use.

**Discussion:**

Respondents either drew upon their social resources and implemented strategies to support behaviour change or experienced social strain and poor mental health in the absence of supportive social strategies. Future research should explore how social networks can impact maintaining non-drinking status and accessing supports. Mixed methods research combining N-of-1, EMA, and egonets can provide novel insights into social dynamics.

## Introduction

Taking a social network focus within an N-of-1 design enables the study of dynamic changes at the micro level as to how individuals experience their social worlds, and the variety of ways this may influence alcohol use. This study aimed to provide insights into the dynamics of the social processes relating to alcohol consumption, applying novel research methods that have not previously been used with people who self-identify as heavy drinkers. The context for the study was the introduction of a Minimum Unit Price (MUP) for alcohol in Scotland, in May 2018. MUP is a policy that sets a minimum price per unit of alcohol that can be sold, the initial minimum price was 50 pence per unit of alcohol. The purpose of this policy change was to reduce alcohol-related harm by making alcohol less affordable.

Social relationships influence health through mediating pathways, including social support, influence, engagement, and access to resources (Berkman et al., 2000). Similarly, having densely connected social contacts who drink is related to more frequent alcohol consumption (Russell et al., 2023), and an increase in the number of network members who are supportive of abstinence is a predictor of reduced drinking (Stout et al., 2012). Interacting with a network of people recovering from dependence may increase *recovery capital* providing resources such as social connection, support, practical strategies, motivation, a sense of meaning, and a positive identity (Best et al., 2015). People who want to reduce consumption may also find it beneficial to break ties with peers who use substances (Dingle et al., 2015). The development of a recovery-focused social identity is theorised to be a key mechanism leading to a sustained recovery from substance use dependence (Best et al., 2016). However, the emphasis on identity overlooks structural determinants such as poverty (Fomiatti et al., 2017), and the dynamic and varied nature of social interactions, particularly among those not strongly connected to a recovery-focused social community, or the difficulties inherent in changing one’s social network transition at the same time as a change in substance use behaviour (Anderson et al., 2021).

Identity and behaviour are influenced by close ties that provide *bonding capital*, characterised by support, co-operation, and effective behavioural sanctions (Burt, 2001). However, weak ties are important also, providing *bridging capital*, access to information and opportunities not available in the immediate social circle (Granovetter, 1983). A healthy network should contain both types of social capital, but recovery is associated with developing additional bridging capital (Panebianco et al., 2016). Individuals who self-identified as heavy drinkers are more likely to have smaller, less diverse networks composed of close ties, where there is less opportunity for participation in different types of social relations (Mowbray et al., 2014).

Social interactions can be explored using N-of-1 methods. N-of-1 explores dynamic changes over time, rather than aiming to determine stable individual characteristics. The questions for an N-of-1 aim to capture within person variability on factors related to the aims of the study. Social networks can also be assessed through egonet interviews that assess social ties and provide information on social structure beyond simply the amount or type of social contact an individual has, and provide the opportunity to analyse the structure of social groups (Crossley et al., 2015). For example, having five social contacts who all know each other is a different social context than having five social contacts who do not interact. Having fully connected social groups may act as a social constraint on the respondent, as this social group may share information and opinions about the respondent. By comparison, having five social contacts who do not know each other may be less constraining, as interactions with one of these contacts are entirely independent of the others (Burt, 2001). A central concept in social network analysis is that the structure of the network can determine individual outcomes by shaping the flow of resources that create access to opportunities and behavioural constraints (Due et al., 1999).

In the current study, we applied an N-of-1 design with a particular focus on social networks to understand the social context of alcohol use at the time of the implementation of MUP; the within-person association between MUP and consumption is reported elsewhere (Kwasnicka et al., 2021). In this study, we explored the role of social networks and social support in greater detail using egonets and exploring alter-alter ties (i.e. relationships among participant’s social contacts). The research questions were: among the target population of the MUP policy (i.e. the population who drink or have drunk to harmful levels) (1) How are social contacts structured? (2) How are social contacts related to alcohol use? (3) How do social contexts influence the experience of MUP implementation?

## Methods

### Design

This study adopted a mixed methods approach that included N-of-1 study with Ecological Momentary Assessment (EMA) (Perski et al., 2022) – smartphone-administered daily surveys followed by social network exploration with an egonet qualitative interview (Chamberlain, 2006). The frequent survey data collection method EMA (Shiffman et al., 2008) holds promise for substance use research as it captures data patterns and trajectories better than retrospective questionnaires (Shiffman, 2009); however, it has been underused in individuals who self-identify as heavy drinkers (Businelle et al., 2024). We used a Delphi workshop and peer feedback to develop the EMA survey questions and study materials, and we employed participatory methods to conduct the study (recruitment and delivery through peer researchers).

#### Participatory approach

The project incorporated elements of participatory research methods (Livingston & Perkins, 2018) working in collaboration with the Scottish Drugs Forum (SDF) peer research team. The SDF peer researchers have personal experience with alcohol and other drug dependence and have undertaken research skills training. Including peer researchers in the study from the beginning provided the opportunity to meaningfully represent the voice of those affected by alcohol dependence in how the research was designed and conducted, within the constraints on the topic and methodology. This approach facilitated the peer researchers’ involvement to sense check, pilot, and revise the study materials, which is of particular importance for an N-of-1 study where pre-existing survey items were not available or appropriate.

#### N-of-1 study

N-of-1 questions were asked daily and referred to the last 24 h, using 0–100 sliding scale, for example, *Over the last 24* *h, how would you rate your mood?* (0 – very down, 100 – very happy). Questions addressed mood, stress, withdrawal symptoms, feelings associated with drinking, motivation, temptation, efforts to reduce or stop drinking, or to ‘stay stopped’. Social context questions addressed personal contacts: *Over the last 24* *h, have you met up with?* (friends, family/partners/children, professional workers, support group peers, others); with question routing to ask: *How did you get on with* (friends/family/professional workers/support group peers/others) *over the last 24 h?* depending on which contact was selected. We also asked: *Did you get any help or support from AA / mutual aid over the last 24 h?* and *Did you get any help or support from drug and alcohol services?* (0 – not at all, 100 – spent almost all day) and *Did you seek help from drug and alcohol services in the last 24 h?* (yes /no). We also asked how much money influenced the amount and type of alcohol drunk and drugs used, and how much time the person spent in situations where alcohol was available. We asked daily about the alcohol consumed and drugs taken – where, what type, and how much consumed/taken. At the end of the survey, we always asked: *Please tell us about anything that affected how you were feeling, or that had an impact on your drinking that we have not asked about* (open text – question not randomised). To our knowledge, this was the first study that used N-of-1 design to explore drinking and social interactions among individuals who self-identify as heavy drinkers.

### Procedure

The study received ethics approval from the University of Glasgow Medicine Veterinary and Life Sciences Ethics Committee (Reference: 200170077) and it was conducted from January 2018 to March 2019. The fieldwork was conducted by peer researchers from the SDF in 2018. Three of the study authors conducted a one-day training workshop with the SDF peer team. The training covered issues such as: how best to administer the study materials and obtain informed consent; how to operate the smartphone and complete the daily survey; how to maintain contact throughout the study: safeguarding and signposting participants to sources of support; and how to conduct the egonet interviews.

#### Recruitment

Participants were recruited via the peer team’s contacts within their local communities including people with a history of drug and alcohol use, and those in contact with treatment services, mutual aid or recovery groups. Recruiters explained the purpose of the study, the voluntary nature of participation, and explained that participants would receive daily texts with a link to complete the online survey. Those agreeing to take part completed a baseline survey and were given a demonstration of the online survey. Participants were offered a smartphone pre-loaded with sufficient data to complete the online surveys if they did not have a smartphone or did not wish to use their own phone. Recruitment took place from rural areas and intermediate-sized towns in an area in the east of Scotland that was not a recruitment site for any of the other research projects studying MUP.

#### Participants

Inclusion criteria for participation included adults who self-identified as heavy drinkers (defined as drinking alcohol at a level that is likely to be harmful to health and wellbeing); or who identified as having stopped or cut down (this included people who perceived themselves to be ‘in recovery’, those who were abstinent, and those who were controlling their drinking). We included current or former heavy drinkers, as the study aimed to understand the different social dynamics involved at different stages of alcohol use, such as differences in network composition between those currently drinking and those who have stopped. Respondents were not recruited if the peer team perceived them as being too intoxicated to provide informed consent, or if they had literacy and language difficulties. Participants did not receive any reimbursement for study participation.

#### Baseline recruitment survey

The baseline survey collected information on gender, age, alcohol and other drug use history and brief information on the extent of contact with family, friends, and the Social Outcomes Index (SIX) Scale (Priebe et al., 2008).

#### Daily survey data collection

Respondents received daily surveys sent directly to their mobile phone or to the mobile phone that they received from the research team. All surveys were set to be delivered daily for 12 weeks at 7 pm. Respondents received the same survey each day with questions randomised within each block and with a comment box at the end. The survey data was set up in Qualtrics (Qualtrics LLC, Utah, USA). The quantitative survey data (excluding detailed social interaction data) is published elsewhere (Kwasnicka et al., 2021).

#### Face-to-face interviews

After all participants had completed the smartphone survey element of the study, they were invited to take part in a face-to-face interview with a peer researcher. The interviews involved completing an egonet social network component (Chamberlain, 2006). At the beginning of the interview, respondents were asked if there had been any change in their alcohol, other drug, or service use status since taking part in the phone survey and before proceeding to the social network data collection. To our knowledge, this is the only study of social networks within the context of alcohol policy change in Scotland.

#### Social network data collection

Respondents were invited to complete an egonet interview: a semi-structured drawing task to collect information about people in their lives during the time they took part in the study. In social network analysis terminology, the participant who is providing the information is referred to as ‘ego’, and others that they have social connections with (about whom they provide information during the interview) are referred to as ‘alters’. The egonet interview involves guiding the respondents through four tasks: (1) *name generator* – to create a list of names of people in the respondent’s social network (alters); (2) *name interpreter* – to assess the characteristics and qualities of the ego-alter relationship; (3) *alter attributes* – to assess characteristics about the alters; and (4) *alter-alter ties* – to determine whether or not alters are connected. All interviews used the same visual prompt, a circle divided into five equal-sized segments labelled as ‘Family’, ‘Working in services’, ‘Friends’, ‘Others I’ve met’ and ‘Others I haven’t met’ with ‘Me’ in the middle of the circle; and with four concentric rings indicating different levels of emotional closeness, those in the centre ring being ‘closest’ and those in the outside ring ‘distant’.

At the start of the interview, respondents were asked to list the names of people they had met during the time they were taking part in the study. The approach used an interaction-based name generator (who they had interacted with recently) but also integrated a position-generator approach (Bidart & Charbonneau, 2011) by using segments to identify people in different social positions, as well as information on social contacts that may be important to the respondent but whom the respondent may not have interacted with recently. The interviewer generated a list of alters in each segment where appropriate, the names were written on post-it notes along with information about the alters’ gender, age, alcohol, other drug use, and substance treatment service use status. Interviewers asked respondents to place the named individuals on the diagram, with the alters that the respondent felt closest to in the centre, and others further away from the centre according to how close they were.

The names or initials of the alters were drawn on the page with a circle around them. Then, respondents drew lines to connect circles if the individuals knew each other with the prompt *If they passed each other in the street, would they stop and talk to each other?* Finally, respondents were given different coloured pens and asked to circle individuals with red or blue according to the level of support they received. *Thinking about staying in good health, feeling good, or using alcohol and drugs: draw circles around people that made things easier (in blue) or made things harder (in red)*. This question accounted for the fact that a currently drinking respondent may have influences on their substance use, while those that had stopped drinking would have influences on how they were feeling about their substance use, but not substance use itself.

At each stage of the interview, interviewers prompted discussion of their choices, e.g. *Why was this person placed as close / very close /distant? Why were they a positive influence or negative influence? How did the respondent know the alter? How did these two alters know each other?* At the end of the interview, respondents were asked if they had heard about the change in alcohol MUP, and whether this had affected their drinking.

In conducting the egonet interviews, the naturally occurring conversations between the peer researcher and the participants provided in-depth information on the nature of the participants’ social relationships and broader social influences on their alcohol use. This interview data was transcribed verbatim and coded using NVivo. The egonet drawings were transcribed using VennMaker software (Gamper et al., 2011) according to a transcription protocol developed by the research team (MA and MMcC). The transcription process also involved listening to the audio recording of the egonet interview and modifying the digital egonet data where appropriate, for example, one respondent had used a red pen to indicate positive rather than negative alters, so this was changed in the transcribed data.

#### Social network analysis

We calculated quantitative measures from the egonet diagrams: (1) number of alters (i.e. people they reported being in contact with during the study); (2) proportion of positive alters (i.e. proportion of positive social connections); (3) proportion of alters drinking a lot (i.e. proportion of social connections who are currently drinking a lot); (4) proportion of alters using drugs (i.e. proportion of social connections who are currently using drugs); (5) total alter closeness (i.e. how many alters, weighted by perceived closeness); (6) ego constraint (i.e. extent of potentially limiting social contact due to alter-alter ties); (7) index of qualitative variation by segment (i.e. diversity of the type of alters mentioned).

Initially, we wanted to combine, compare and interpret data from N-of-1 study (Kwasnicka et al., 2021) with the egonet results. However, due to low participation rates in the egonet interviews, we have not had enough data to compare the results using these two methods. We drew on principles from Qualitative Structural Analysis (Herz et al., 2015) to integrate analysis of network maps and interviews, with a primary focus on the analysis of the interview.

During the interviews, some respondents did not wish to disclose information about their substance-using friends. The interviewers handled this by discussing their friendship networks, in some cases this was accompanied by an approximate number of friends within the group, but in others it was not. When the network data was transcribed into VennMaker, the specified number of friends was encoded for those alters, and where it was not specified, three alters were used, hence reflecting a potential underestimate of network size. The three alters were assigned characteristics according to how they were described in the qualitative recording (e.g. all currently drinking a lot, a mixture of men and women).

#### Qualitative data management and analysis

All interviews were audio recorded and transcribed verbatim by professional transcribers. Interviews had a mean duration of 34 min and a range of between 20 and 62 min. All respondents were given numbers and pseudonyms to ensure their anonymity. We used Nvivo software (QSR International Pty Ltd) to facilitate data storage retrieval, coding and analysis. Each transcript was read repeatedly by the coding team (AOG, MMcC and MA) and the main themes were identified deductively (based on the research objectives) and inductively (based on themes that emerged during analyses). Codes were agreed and entered into NVivo. All coders coded a sample of the transcripts and outputs from these broad codes were read and discussed by all coders before the remaining transcripts were coded. Once the transcripts were coded in line with the themes, the content within each theme was analysed and emerging themes across and within cases were noted using framework matrices. Raw data in the form of direct quotations were highlighted in the matrices. This Framework Analysis method facilitated the comparison of data across and within individual cases and allowed visual recognition of patterns (Rosen et al., 2023). This iterative process enabled inspection of the data and identification of consistencies across the themes and individuals, as well as atypical cases.

## Ethics statement

Ethical approval was granted by the ethics committee for the College of Medicine, Veterinary and Life Sciences, University of Glasgow (Ref: 200170077).

## Results

Six participants took part in egonet qualitative interviews (IDs 1009, 2003, 2004, 2007, 2011, 3007). Fifteen respondents had provided sufficient EMA data regarding social contact (Table 1), however, only three of these agreed to take part in egonet interviews (IDs 1009, 2004 and 2011).

**Table 1.** Baseline characteristics of study participants.

ID	Gender	Age	Other substances+	Stopped or controlling alcohol use	Employment status	Accommodation status	Living alone	Recent social contact	Social Outcomes Index
1002	Male	46–50	Co-codamol	Yes	Not working	Independent	Yes	No	3
1003	Male	46–50	None	No	Employed	Independent	No	Yes	5
1004	Female	55–60	None	Yes	Employed	Independent	No	No	4
1005	Female	50–55	Cannabis	No	Not working	Independent	Yes	Yes	4
1006	Male	36–40	None	Yes	Employed	Independent	Yes	Yes	6
1007	Female	40–45	Cannabis	Yes	Not working	Independent	Yes	Yes	4
1008	Female	55–59	Alcohol	Yes	Not working	Independent	Yes	No	3
1009*	Other	50–55	Alcohol	Yes	Not working	Independent	Yes	Yes	4
1011	Male	40–45	Alcohol	Yes	Not working	Independent	Yes	Yes	4
1012	Female	46–50	Alcohol	Yes	Volunteer	Independent	Yes	No	4
1013	Male	50–55	Alcohol	Yes	Volunteer	Independent	Yes	Yes	5
2001	Male	56–60	None	No	Not working	Homeless	Yes	Yes	2
2003*	Female	60–65	Alcohol	Yes	Not working	Independent	No	Yes	3
2004*	Male	66–70	None	No	Not working	Independent	No	Yes	3
2005	Male	36–40	None	Yes	Employed	Independent	Yes	Yes	6
2006	Male	50–55	None	Yes	Employed	Independent	Yes	Yes	6
2007*	Male	60–65	None	Yes	Not working	Independent	Yes	Yes	4
2008	Female	40–45	Valium	No	Not working	Independent	No	Yes	3
2009	Male	36–40	Heroin	No	Not working	Independent	No	No	2
2010	Male	25–30	None	No	Not working	Homeless	Yes	Yes	2
2011*	Male	60–65	Dihydrocodeine	Yes	Not working	Independent	Yes	Yes	4
2017	Female	40–45	None	Yes	Not working	Independent	Yes	Yes	4
3003	Male	30–35	None	Yes	Not working	Independent	Yes	Yes	4
3006	Male	30–35	None	No	Not working	Independent	No	Yes	3
3007*	Male	16–20	None	No	Not working	Independent	No	Yes	3

Note*.* Study participants with * took part in egonet interviews. + - None denotes alcohol was the only substance, alcohol denotes a different primary substance of dependence.

### Social network analysis

Respondents who completed egonet interviews reported 10.8 social contacts on average during the time they took part in the study and rated about half of their networks as positive supports (Table 2). Approximately 10% of each respondent’s networks were perceived as ‘drinking a lot’, as opposed to ‘drinking a little’ or ‘not at all’. There was a higher proportion of drinkers in the networks of participants who reported they were using alcohol than in those who were abstinent. The Index of Qualitative Variation was slightly higher for those who had stopped drinking than the drinking group; this index measures how many categories of individuals (family, friends, professionals, etc.) were present in the social networks.

**Table 2.** Social network measures by drinking status at recruitment – number and proportion of alters.

Network measure	Currently drinking mean (SD)	Stopped drinking mean (SD)	Overall mean (SD)
Number of alters	12.5 (2.12)	10 (0.82)	10.8 (1.72)
Rated positively	0.5 (0.71)	0.5 (0.46)	0.5 (0.47)
Drinking a lot	0.2 (0.25)	0.1 (0.06)	0.1 (0.14)
Using drugs	0.4 (0.56)	0.2 (0.18)	0.3 (0.3)
Total closeness score	45 (5.66)	30.8 (6.85)	35.5 (9.42)
Network constraint	0.29 (0.08)	0.25 (0.07)	0.3 (0.07)
Diversity of alter types (Index of Qualitative Variation)	0.5 (0.2)	0.8 (0.1)	0.7 (0.19)
**Respondents**	**2**	**4**	**6**

**Figure 1.**
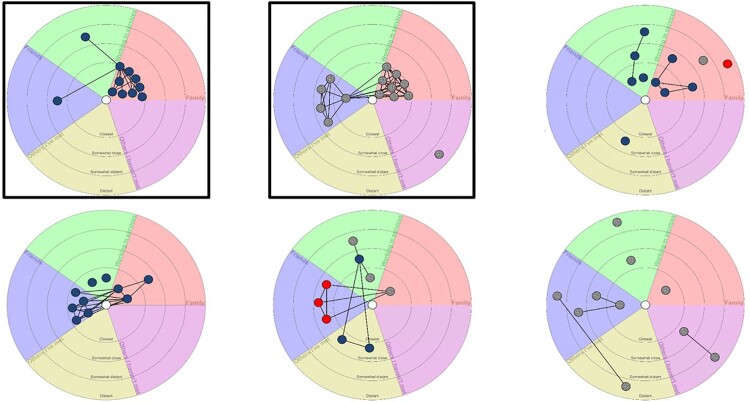
Anonymised egonets for six respondents. Note. Figure 1 shows digitised and anonymised versions of the egonets completed by the six interview respondents. Images are ordered in reading order (from left to right, then left to right on second row) according to the increasing index of qualitative variation for each segment. Those who placed alters in more segments of the egonet diagram – and thus had more variation in their social network, appear towards the bottom right. Top row IDs 2004, 3007, 2007; bottom row 2003, 2011, 1009. ‘Current drinker' sample highlighted in square frame. Blue denotes a positive alter, grey denotes neutral, and red denotes a negative alter. Egonet segments denote, clockwise from North: professional in health/social care services (green), family (pink); others that I have not met (purple); others I have met (yellow); friends (blue). Participants 1009, 2004 and 2011 provided sufficient EMA data to assess and to illustrate their social contact over time.

Qualitatively, there were important dimensions of social relationships that underpinned the networks. Some respondents spoke about the high levels of relational strain in the past, which meant that some family members were not represented in the networks. This historical context had an important role in structuring the diversity of contacts in the network (Figure 1).

The ‘current drinkers’ – respondents who were not stopping or reducing their alcohol at the time of baseline recruitment – are highlighted with a square border. These two respondents had the least diverse social networks, with a predominance of family contacts, or family and friends only. In both cases, they perceived their family or friend clusters of social contacts as being close to them, and all members of these clusters as being similarly close. This pattern of perceived close clusters appeared for one of the ‘stopping drinking’ participants, while the remaining three showed greater diversity in placing social contacts in terms of closeness. This may reflect a greater diversity of social interactions, but also some evidence of social strain, such as the family member perceived as a negative influence and placed at a distance for the top right panel (ID 2007). There is an observable pattern of dense clusters of close ties in the networks of drinking participants and more sparsely connected weaker ties in most of the networks of those who had stopped drinking. Social contacts tended to be viewed as having positive influences on people’s wellbeing and alcohol use, with only two respondents mentioning negative connections. In one case, this was a strained family relationship (ID 2007), and in the other case related to a cluster of friends who had negative influences in relation to drinking (ID 2011).

### Visual plots of social contact over time

The figures below show the extent of social contacts the respondents reported during the EMA study. Time moves from left to right, and each horizontal row refers to each category of social contact the respondents answered about (family, friends, peers, etc.). The coloured lines indicate that the respondent did meet someone from the relevant group on the day in question, while the dark grey background shows that they did not. The light grey vertical lines show the days where the participant did not complete the survey.

**Figure 2.**
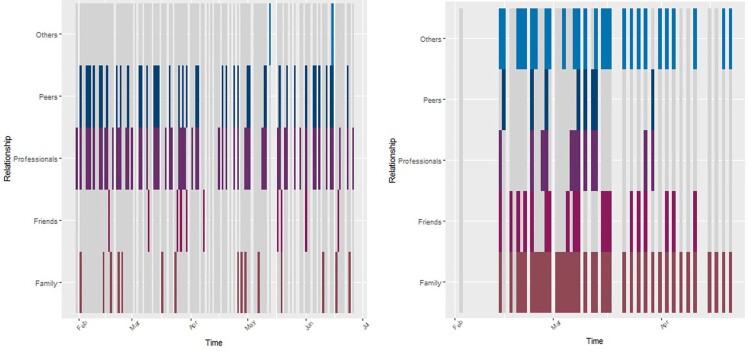
Social contact over time - ID 1002 and 1004

Figure 2 shows the social contacts for participants 1002 and 1004. The participant 1002 was in the study for 166 days in total, and answered on 77% of days. The blue and purple rows show that they were in contact with professionals almost every day they completed the survey, and drug recovery peers on most days. They were much less frequently in contact with family, and less frequently with friends outside the recovery community. For participant 1004 who was in the study for 100 days in total, and answered on 67% of days, in contrast to 1002, this respondent was in contact with family almost every day, was in contact with professionals and peers less regularly, much less frequently in April than in March, and was very frequently in contact with other non-family/friend/peer contacts. The remaining figures (Online Supplement 1) show the response patterns for those that provided moderate or good quality data – there is clear variation in the diversity and the frequency of social contact between the respondents. The remaining plots are presented in small multiples to facilitate visual comparison, for instance, 2001 and 2005 had no family contact, but regular contact with friends and 2006 and 3003 had frequent interactions with a diverse range of people.

### Thematic analysis of interview data

The thematic analysis of the egonet interviews provided further insight into the participants’ social networks and broader social influences on their drinking behaviour. This data demonstrated the richness that qualitative interviews add to our understanding of behavioural change around alcohol. The deductive and inductive analysis of the interview data identified four key themes that influenced drinking behaviours – peer and family networks; the experience and impact of stress; motivation levels; coping strategies – within the context of the social world they inhabited.

#### Peer networks

In seeking to transition from being a heavy drinker to a controlled or non-drinker, respondents related that they had drifted away from family and friends associated with their drinking and as a result tended to have more diverse but less close social contacts. Instead, they connected with groups that implicitly or explicitly supported their abstinence, such as a church group or peers from local alcohol/drug services or mutual aid support services. These networks provided companionship and helped fill the vacuum in respondents’ social life in the absence of alcohol. The respondents found it valuable for maintaining behavioural change to be able to talk to someone who had the experience of controlling their alcohol use and with whom they could discuss strategies for avoiding alcohol and coping mechanisms when they felt tempted to relapse. For example, Gayle (ID: 2003, all names used throughout the results section are pseudonyms) reported that her local service, ‘*keeps me grounded to what I am doing … when I come here I’m totally honest about what I drink*.’ Neil (ID: 2007) described how he found these groups helpful: ‘*they gave us tools … I’ve met a lot of people that made me realise, I’m not on my own*.’

The availability and geographical proximity of peer and service support networks were important for social contact. Jackie (1009) reported her frustration of seeking but not being able to access the support she wished, and felt lonely and isolated as a result as she had no other contact with family members. She related that, ‘*I don’t go out as much now because I feel that I haven’t got the social contact I had before*.’

Ken (3007) was one of two interview participants who had continued drinking during the study. He was adamant that his social network did not influence his alcohol use, but this was a unique response. A recurring theme in the respondents’ narratives was the influence of their existing social environment and networks on their attempts to change their drinking behaviour. For example, Iain’s (ID: 2011) account demonstrates the potential of social networks to have a negative influence on drinking behaviour. In his case, he had stopped drinking completely but after a hospital admission returned home and resumed drinking which he attributed to being unable to leave the house due to a heavy snowstorm and being visited by his drinking friends who brought alcohol with them. However, though he maintained contact with his drinking friendship network and continued to drink with them he maintained a change in his alcohol consumption and refrained from drinking the higher strength alcohol his friends consumed:

They all seem to drink the stronger stuff at night … I don’t drink the same as them. I wouldn’t ask them for a cut of their stuff because I just drink my own stuff all the time. I just like sticking to the same drink.

#### Family networks

Family relations were found to be a key influence on drinking behaviour. Bob (ID: 2004) continued to drink during the survey, ‘*a half bottle of whiskey per night … I’m a creature of habit*’. He described a close family network from whom he derived strong social support though, ‘they hardly drink’. Neil (2007) reported strained relationships with some family members and his children. However, he derived strong support from the rest of his extended family which enabled him to cease drinking. For example, his family had alcohol-free family gatherings to help support him:

I’m lucky, I’ve got a strong family that support me, and don’t turn their backs on me. Even when I do relapse, they’re there to give me a kick up the backside, they’re honest with me. And I really appreciate that.

He related as an example his sister’s support for his abstinence at a family gathering:

One of the family who didn’t know about my drinking, distant family that was there, actually had put a drink down in front of me. My sister quickly got it out the way.

In contrast, Iain’s (2011) relationships with his familial network – a large extended and blended family – were mainly negative though he maintained that this had little impact on his drinking, ‘*I never see them. Out of sight, out of mind, kind of thing*’.

The need for social support systems to help maintain behavioural change varied across the cases. Some respondents found family and peer support a great resource, others expressed a desire to cope on their own even though this meant increased levels of isolation. The distinction appeared to lie in whether their social networks were a positive or negative influence on their drinking behaviour and enabled drinking or abstinence. For example, conflict with family and friends could spark a change in drinking habits. As Ken (3007) related: ‘sometimes when I’d been arguing I obviously did kind of go out and drink more’. And, as Jackie (1009) explained: ‘*I keep myself to myself because it’s safer … I don’t really have much to do anybody else*.’

#### Experience and impact of stress

The respondents’ narratives depicted high level of historic and current stress derived from their own or family members’ ill health, conflict with family and/or friends, and experience of trauma. Their accounts demonstrated the numerous challenges they faced both during the study period and over their life course, and how this impacted on their alcohol consumption, but also their many attempts to find coping mechanisms to deal with these stressful situations.

For example, Gayle (2003) sought to manage everyday stress so that it would not affect her alcohol intake. Her coping strategy was to ‘*set a limit of what I drink. I buy that set limit and that’s it*’. However, during the study, she experienced high levels of stress due to sister’s illness which impacted on her drinking:

My last two days have been horrendous … my alcohol consumption has gone up in the last two days … I wandered round the Co-op about nine o’clock last night to get a bottle of wine on top of what I already drink.

Previously, an extremely stressful social situation with her former partner had also affected her alcohol intake: ‘*My ex-husband was there, the one that used to beat me up and everything, so I was already anxious and I lost track of what I was drinking*.’

#### Motivation

Amid such stress, staying motivated was an ongoing challenge for the interview participants. For Neil (2007), health concerns, ‘*pride*’ and not wishing ‘*to let his family down*’ were important motivating factors to both stop alcohol use and withstand the temptation to drink at trigger events such as football matches and at a family funeral. Practical issues such as sleeping better provided motivation for change also.

I’ve done quite a bit of damage to myself, and if I keep going, it’s only going to make it worse, to the point I won’t recover, health wise. … I’ve realised that I have to do it. [And] pride in myself … [at a family gathering] I found it, not easy, but I was able to control myself, and drink orange juice, all day. I was proud of that fact, and so was my sister. She was really proud of me, she told me that a few weeks after, that I managed, and kept away from the alcohol.

#### Coping strategies

Respondents’ narratives indicated a wide range of coping strategies they had developed to maintain their change and reduce and/or stop their alcohol use, many of these involved getting support from their social contacts. Many referred to these strategies as *‘*the tools’ [a term used by self-help and mutual aid groups] and included distraction methods such as going for a walk, or playing a game of golf. For example, Bob (2004) played golf with his brothers; Gayle (2003) treated herself to a ‘fish supper’ as a treat after attending her self-help group. Neil (2007) described a range of coping strategies he employed such as keeping himself active, busy, cooking, and deferring alcohol use until later in the day. Neil derived a high level of satisfaction from being able to control his drinking and this helped him to sustain his behavioural change. Over the duration of the study, Neil had started and then stopped drinking, he recounted how he had achieved this:

Like the first time [he had stopped drinking during the survey period] I started using the tools. And I went, right, if I can reduce it, start later in the morning, and reduce it for the evening, until eventually, over time … I stopped drinking … I tend to use family a lot … and if they’re not available, then I tend to go out … just jump on a bus and go somewhere that I know I can stay away from the alcohol.

Iain described reducing their alcohol use gradually over time, they hoped this would alleviate the withdrawals they had experienced previously. Jackie (1009) who reported feeling isolated and lonely put in place strategies to cope including proactively seeking support from a small number of friends/neighbours who were not drinkers and walking her dog. She related, that she now has ‘*my wee dog and I’ve got, you know, a wee social life there. I think that helps*’.

The availability of alcohol in the participants’ social environment was a common and constant challenge to address. For example, respondents with partners who drank alcohol found strategies to cope with this temptation as Neil (2007) related:

As time went on, I got used to it, and it didn’t bother me. It didn’t cross my mind, I just got on, either made myself something to eat, and a cup of tea or coffee. And I still chatted away, even though there was a glass of wine sitting in front of me, the partner’s, it didn’t bother me, after a while. Because I got used to that, and I used the tools I had learned, to block that from bothering me … Because I knew, if I let that bother me, then I’m on that downward spiral again. I had to keep myself busy.

#### Impact of MUP

The cost of alcohol, including the MUP-related rise in cost, did not appear to have much influence over patterns of alcohol consumption among interviewees. Depending on their choice of alcohol, some found no difference in the price after the implementation of MUP and said that ‘*nothing’s changed*’. Two respondents reported finding alternative supplies for the white cider they preferred and were able to purchase at the old price. Neil (2007) related:

I was getting it in different ways … There was always a way to get the reduced price … And to be honest, it’s quite surprising how easy it is, to find these outlets.

All data pointed to the alternative influences on their drinking behaviour noted above and also being busy, bored, having or not having money, feeling tempted and guilty for drinking or feeling strong for withstanding temptation. These feelings and thoughts were seen to change day by day reflecting the fluctuating influences and challenges faced by people seeking to change their alcohol consumption patterns.

### Data triangulation

Triangulating the EMA, network map, and interview data shows consistency across the data captured by each method. Current drinkers described social constraint and/or isolation that was mirrored in the high social closure visible in their networks, and low social engagement in their EMA data. For example, Jackie (1009) described low social support and felt safer staying isolated to avoid relapse. This is reflected in the network map where the absence of many close ties indicates a deficit of bonding capital. Her EMA data depicts social isolation punctuated by infrequent social contact. Stopped drinkers tended to describe more varied social activities, consistent with the reduced closure apparent in their networks. For example, although Iain (2011) had pro-drinking influences in his network, he was able to maintain reduced drinking. His network shows his negative drinking peers are balanced by a separate cluster of mainly positive peers from services, with no overlap between the two groups. His EMA data confirms this diversity of social contact. EMA data is also useful for showing social isolation not readily apparent from interviews or maps. Bob (2004) describes strong family support and his networks is mainly a large cluster of close family ties. This could suggest strong bonding capital; however, the EMA shows two months with almost no social contact. A more diverse network could provide more varied socialisation when the family is not available. Integrating all three data sources paints a more complete picture of the social lives of participants and how their social environment can affect their drinking (Table 3).

**Table 3.** Triangulation of data from interviews, networks, and EMA data.

	Interview theme	Network feature	EMA feature
1009	Limited support/socialisation. Stays isolated to avoid relapse. Recovery peers provide some socialisation.	Sparsely connected weak ties with few close ties, all neutral. Indicates limited bonding capital.	Infrequent social contact and long spells without any social contact.
2003	Can be honest at self-help groups. Alcohol increases with stress and anxiety, caused by family issues.	Densely clustered close ties; no weak ties. All positive ties. Lack of bridging capital suggests difficulty avoiding stressors in immediate circle.	Insufficient EMA data.
2004	Support from a close family network. Coping mechanisms include golf with brothers.	Predominantly close family members. All positive influences. Bonding capital.	Regular family/friend contact then almost no social contact for two months.
2007	Self-help groups, identifying with others in recovery. Family support with minor strain. Strongly motivated. Successful utilisation of coping strategies (‘tools’).	Mix of strong and weak ties. Mainly family and services. Mostly positive, one negative. Nobody in ‘friends’ segment suggests broken ties replaced with groups/family.	Insufficient EMA data.
2011	Social influence caused relapse but reduced drinking. Family described as distant, negative.	Clique of negative friends. Only one family alter. Mainly close ties. Structural hole between services and friends/family.	Frequent, diverse social contact. Friends, family, peers, professionals.
3007	Claimed no social influence but later admitted he’d drink more after arguments.	Two dense clusters of close ties (friends, family). One distant isolate who hasn’t been met. All neutral influence.	Insufficient EMA data.

## Discussion

Based on this integrated data, we found that social and interpersonal relations (particularly with family members) and social context were key factors relating to alcohol use. Respondents either implemented strategies to support behaviour change that drew upon these social resources; while others experienced social strain and poor mental health – in the absence of supportive resources – which placed them at risk of increased alcohol or other drug use. This study employed novel methods to provide a rich understanding of the factors that influence individuals’ drinking on a dynamic basis, and how these are influenced by MUP and their social environment. The N-of-1 approach helped identify between individual differences that are masked using a group-based statistical approach, and which can triangulate information on the idiosyncratic experiences reported qualitatively. We provided further information about daily changes in consumption, contact with services, and substitution to other substances. Social network analysis and qualitative accounts facilitated the exploration of contextual influences of policy change. This complements the statistical information on wider population trends in relation to MUP (Beeston et al., 2019).

The social network information collected during the interviews showed a high level of variation in the degree of social support, strain and connectedness. These factors can all influence health and wellbeing (Berkman et al., 2000), and in relation to dependent alcohol use, they are key features of the social environment that are potentially modifiable, and which may act to mitigate the influence of psychosocial factors – for example, to reduce stress or offer alternative spaces for social interaction where situational availability of alcohol is lower.

There is some indication that the current drinkers’ social contacts involve dense clusters of close ties compared to the weaker ties and more diverse social contacts among the stopping networks. This suggests that the structure of social contacts among current drinkers may facilitate bonding capital but lack the bridging capital and reduced social closure that can help sustain recovery. This would be theoretically consistent since problem drinking is associated with low network diversity (Mowbray et al., 2014) and abstinence is associated with the increased diversity offered by weak ties (i.e. a looser network of social contacts) (Panebianco et al., 2016).

The presence of negative ties and more open social networks in the stopping networks suggests that stopping can create a relational strain with network members who continue to drink or use other drugs. These dynamics may lead to the desire for an abstinent network (Best et al., 2016). The absence of negative ties for current drinkers may be a feature of their lack of weak ties, it is feasible that relational strain has broken ties until only those connected through obligation remain (Burt, 2001). There is qualitative evidence that structural barriers can hinder the ability to develop new social identities and such efforts are subject to wider socioeconomic and place-based constraints (Fomiatti et al., 2017). Analysis of larger ego network samples would be necessary to confirm these theoretically plausible explanations of the network structures.

The interview data provided additional insight into participants’ social worlds and the adverse contexts that underpin their harmful drinking and the challenges these pose to their attempts to change their drinking behaviours. Additionally, we demonstrated that new methods of exploring day to day changes and social networks (N-of-1, EMA and egonet interviews) are feasible to use in research with individuals who self-identify as heavy drinkers. However, comparable to studies in the general population, not all study participants are willing to engage in all study components, often resulting in incomplete dataset, especially if researchers are aiming to overlap study data from multiple sources and methods used.

### Study strengths and limitations

This is a unique use of an N-of-1 design to look at how social contact relates to alcohol use, integrating data on dynamic changes in daily social contact, egonet social network data and data from qualitative interviews. This study was the first to employ such a novel and broad variety of research methods with individuals who self-identified as heavy drinkers.

The design used in this study was innovative in terms of combining N-of-1 study with the follow up social network interview; however, not all respondents provided sufficient quantitative data, limiting the ability to fully explore the relationship between EMA patterns and social network influences discussed in the interviews. The fact that such a rich, high-quality data was gathered for some of the respondents can be attributed to the engagement skills of the SDF research team and the importance of a participatory approach to research.

Using EMA design, we characterised variability in social contact over time, and evidenced high variability in social contact patterns, from regular daily contact with a range of family, friends, and professionals, through to less frequent, but regular, contact with others, through to near complete social isolation. Regular social contact, in particular for those living alone, may provide an important social routine that confers psychological benefits, even if the frequency of contact is much lower than for those who have co-resident family or frequent peer interactions.

Another limitation of the study is that the EMA data related only to dynamic changes in contact with people in certain social positions (friends, family, professionals, etc.) while the egonet interview data related to named individuals. This was necessary to keep the EMA survey short, as it contained many other questions besides social contact (reported elsewhere: Kwasnicka et al., 2021). A promising area for future research would be to implement a name-generator EMA survey, that could then be directly compared to the names from egonet and qualitative interview data and begin to study changes in dynamic social contact in greater detail.

## Conclusions

To complement traditional sample-based studies of factors related to the change in alcohol use at the population level, N-of-1, social network and qualitative studies can help contextualise the circumstances that may lead to differentiated levels of change. Social context and stressful social interactions in people’s daily lives were factors to explore as points of action to reduce alcohol harm. N-of-1 methods can support the study of dynamic changes in the contexts and environments that people inhabit. This is a promising area for further research to better understand the dynamic nature of people’s social worlds, and also for developing interventions that consider which aspects of an everchanging social environment should be the focal points for supportive change, or preventive action.

## Data Availability

Anonymised quantitative data is available via the Open Science Foundation (osf.io/ESW4D), qualitative information has been removed to prevent inadvertent disclosure of places, participants, or people in the participants’ social network.
